# An Investigation about the Influence of Bleaching on Shear Bond Strength of Orthodontic Brackets and on Enamel Colour

**DOI:** 10.5402/2012/375849

**Published:** 2012-03-22

**Authors:** Isabell Immerz, Peter Proff, Piero Roemer, Claudia Reicheneder, Andreas Faltermeier

**Affiliations:** Department of Orthodontics, University Medical Centre of Regensburg, Franz-Joseph-Strauss Allee 11, 93042 Regensburg, Germany

## Abstract

The aim of the study was to investigate the effect of bleaching on the colouration of tooth enamel and shear bond strength of orthodontic ceramic brackets based upon current whitening practice. The bleaching and bonding techniques were performed on extracted bovine teeth for the investigation of their colorimetric spectrum and the adhesive bond strength on surface enamel. One group was designated as the control group with no pre-treatment. Another group was treated with a 45% hydrogen peroxide solution prior to bonding. The difference in colour was expressed as the Euclidian distance Δ*E*. The resulting shear bond strength was analyzed and evaluated by scores of Adhesion Remnant Index. Statistical analysis was performed using the Kruskall-Wallis and post-hoc test. The colorimetric analysis revealed statistically significant differences between original and bleached as well as bleached and debonded teeth setting off a blue colour shift. Furthermore, statistically there was no significant difference noted in bonding strength between non-treated surfaces and those treated with peroxide. It can be concluded that peroxide pre-treatment does result in colour differences of teeth. Bonding and debonding procedures seem to have no statistically significant influence on the enamel colour using current materials.

## 1. Introduction

The increasing demand for an improved dental appearance, regularity, and colour [1] gives greater importance to the adherence of dental recommendations. Progress has been made in orthodontic brackets with the development of attractive materials and functional design. Bleaching techniques have improved dramatically in an increasing range of products for both dentist-supervised and patient-administered nightguard bleaching applications.

The colour of teeth is influenced by a combination of the light scattering, absorption properties of the enamel and the presence of any extrinsic organic material built up on the enamel, surface especially in areas that are less accessible for daily dental care. Discolouration is often promoted by smoking, dietary intake of tannin-rich foods, and the use of certain cationic agents such as chlorhexidine or metal salts such as tin and iron [2, 3]. Tooth colour can be improved by a number of methods and approaches including whitening toothpastes, professional cleaning by abrasive action, internal bleaching of nonvital teeth, external bleaching of vital teeth and dentist microabrasion of enamel [3, 4].

The scope of this study is restricted to external bleaching of vital teeth carried out by peroxide concentrations as used in office. There are three fundamental bleaching techniques in practice today ranging from in-office, dentist-supervised, to mass market products [5]. The latter contain relatively low levels of whitening agents that are self-applied (i.e., strips). Nightguard bleaching products are also available with higher concentrations of hydrogen peroxide which is then applied to the teeth via a custom-made mouth guard [2]. In contrast, in-office bleaching is generally based upon high concentration levels of peroxide for shorter application periods [6]. Soft tissue must be protected in these treatments due to their sensitivity [7].

Organic compounds that possess extended conjugated chains of alternating single or double bonds are the agents that stain the enamel [5]. Bleaching destroys the double bonds of carbonyl and phenyl rings to a certain extent by cleaving the conjugated chain or by oxidation of other chemical moieties in the chain [3].

Hydrogen peroxide may be applied directly or produced in a chemical reaction from sodium perborate or carbamide peroxide [3]. As peroxide diffuses into the tooth, it strongly oxidizes through the formation of free radicals, reactive oxygen molecules, and hydrogen peroxide anions [8]. The result of the bleaching procedure depends principally on the concentration of the bleaching agent, the ability to reach the discoloured sites, and the degree of peroxide exposure time. Extended duration and frequency that the agent is in contact with the organic molecules provide similar bleaching results as highly concentrated, short-term power bleaching [3].

The chemical composition of adhesives determines to a large extent their adhesive performance. Irrespective of the number of bottles, an adhesive system typically has resin monomers, curing initiators, inhibitors or stabilizers, solvents, and sometimes inorganic filler of which each one has a specific function [9]. New bonding potential and chemical processes have been introduced in the year 1985 [1] using direct orthodontic bonding by means of etching the tooth surface with phosphoric acid.

The structural change in the enamel layer caused by bleaching agents and etching acids is generally not well investigated [5]. Residual adhesive, resin tags are associated with the debonding of orthodontic brackets and which may be affected by prebleached teeth [10].

The aim of this study was to observe the changes in tooth colour and shear bond strength associated with debonding and/or bleaching processes.

## 2. Materials and Methods

Freshly extracted bovine incisors were used with no visual discolouration, obvious grooves, or fissures. Preparation by grinding was avoided as bovine teeth are large enough to provide consistent enamel surface of sufficient area. The teeth were polished, cleaned, and kept in a sodium chlorate solution prior to embedding. To ensure consistent and repeatable positioning of specimens in testing machines, teeth were set in cast blocks (length/width/height: 4/3/5 cm). Only the sound labial surface was exposed.

A total of 100 bovine teeth were randomly divided in 2 experimental groups and treated according to the manufacturer's recommendations.

- Unbleached group: 50 teeth were stored, embedded in the cast blocks, in 0.1% Thymol solution for five days. In preparation for the bonding process, the teeth were rinsed and air-dried. The bonding surfaces were etched with a 20% phosphoric acid gel (Gluma Etch 20 Gel, Heraeus Kulzer GmbH, Hanau, Germany) for 20 s and again rinsed with water and air dried.
- Bleached group: 50 teeth were bleached by the placement of 45% carbamide peroxide (Opalescence, Quick 45% PF, pH = 6.5; Ultradent GmbH, Munich, Germany) around the dried enamel surface for 30 min. In advance to bonding a 0.1% Thymol solution was also used as storage media in this group. The preparation for the bonding process took place as previously described in Group I.

The acid-etched teeth of both groups were primed (Transbond XT Primer, 3 M Unitek GmbH, Perchtoldsdorf, Austria) and then light-cured for 15 seconds. Ceramic brackets (Clarity SL, 3 M Unitek GmbH, Perchtoldsdorf, Austria) were bonded after application of the adhesive (Transbond XT, 3 M Unitek GmbH, Perchtoldsdorf; Austria) and light-polymerized. The bonded teeth were then stored in distilled water for eight days to simulate the salivary oral conditions.

**Measurement of Shear Bond Strength**

Specimens were stressed to failure, shear strength, in a universal testing machine (Instron 5542, Instron Structural Testing Systems GmbH, Pfungstadt, Germany) at a crosshead speed of 1 mm per min. The critical maximum stress prior to failure was determined for statistical analysis. The SBS was evaluated using the formula: *σ* shear = Fmax/A bracket base surface (MPa).

**ARI-Score**

The adhesive remnant index was visually classified for all of the specimens by the use of a spectrophotometer. The ARI is scored: 0 to 3 (0 = no adhesive on enamel, 1 ≤ 50% adhesive on enamel, 2 ≥ 50% adhesive on the enamel, 3 = 100% adhesive on enamel).

The clear impression of the bracket base design guaranteed in most cases a high level of good-quality results. The remnant bonding material was then removed and the enamel polished until no adhesive was visible.

**Colorimetric Assessment**

**Figure 1:**
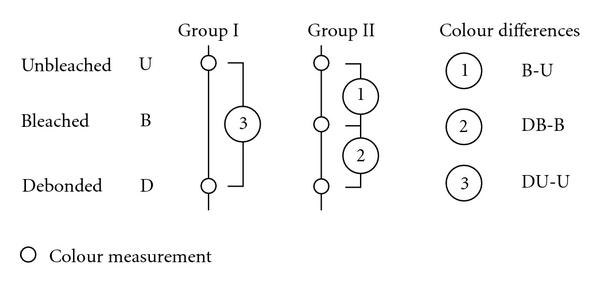
A total of 100 bovine incisors were divided into a control Group I (*n* = 50) and a Group II (*n* = 50). Colour measurements captured colour data of unbleached, bleached, and debonded teeth status.

The Group I, unbleached, colour measurements provided two data sets (data pairs) consistent with the original and debonded condition (Figure 1). The Group II, bleached, three colorimetric recordings were taken (data triplets) in the midfacial location prior to bleaching subsequent to bleaching and after debonding. Colour differences are measured as the Euclidian distance (Δ*E*) in three-dimensional space [11, 12].

Human perception (*E*) assumes that identical spatial differences represent identical differences in colour [13]. The Δ*E* value provides no information on the direction of the colour difference. Consistent with human perception of the colour scale, lightness as well as the colour parameters a* (red-green) and b* (yellow-blue) were also captured.

**Statistical Analysis**

Statistical analysis of the colour data calculated the difference in the individual tooth data pairs (Group I) and triplets (Group II). Testing was accomplished in a successive two-series event. Data sets that resulted in a Δ*E* value greater than three were excluded for clinical reasons.

Statistical analysis was performed by the program SPSS for Windows 12.0 (SPSS Inc., Chicago, IL, USA) using the Kruskall-Wallis and post hoc test. Means and standard deviations were calculated. The significance level was set at *α* = 0.05.

## 3. Results

**Figure 2:**
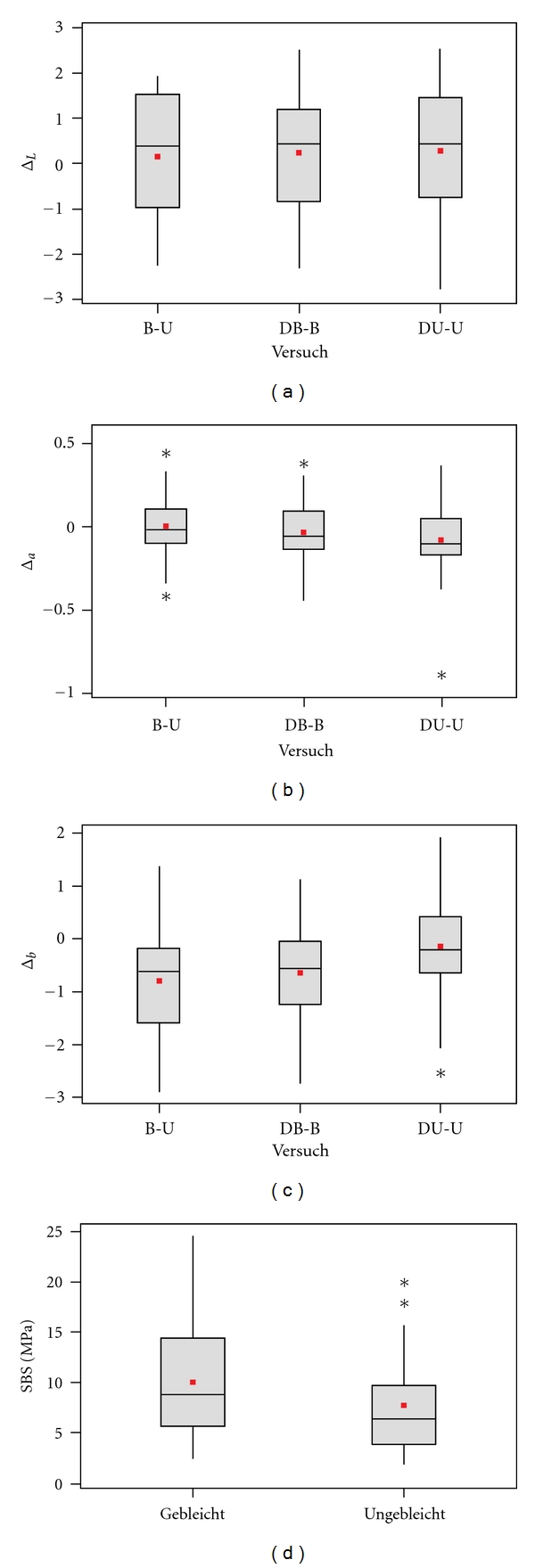
2(a)–2(c) Spectrophotometrically evaluated tooth colour. The difference in colour is expressed by the parameter for lightness (L*), red-green (a*), and yellow-blue (b*). The three test series displayed as box-whisker plots represent the difference between bleached and unbleached (left), debonded and bleached (middle), and plus debonded and unbleached (right). 2(d) Shear bond strength of ceramic brackets bonded to enamel after a 20% phosphoric acid etching for 20 s. Results are displayed for Group II (left) and Group I (right) in MPa.

Analysis of the brightness parameter L* did not result in any significant differences associated with either bleaching itself or debonding (Figure 2(a)). The red-green parameter a* evidenced a slight green shift (Figure 2(b)) for unbleached teeth after debonding established through a one-tailed test and assuming that discoloured teeth tend to have a reddish brown touch [14].

The statistical tests reflected a significant difference for Group II, yellow-blue parameter b*. These specimens, bleached and debonded, had a blue colour shift (Figure 2(c)). There was no measurable blue trend evident within the control group of unbleached and debonded teeth.

Statistical evaluation of the data obtained from the shear test, expressed in MPa, indicates a 0.5 MPa higher shear bond level for bleached specimens (Figure 2(d)). The significance was confirmed by a one-tailed test and considering the Welch-Approximation.

Examination of the debonded sites revealed a strong adherence of the remnant adhesive to the enamel surface resulting in an ARI Score value of 1. A clear impression of the bracket base design was typical in the decision making relative to the scoring process.

## 4. Discussion

The purpose of this study was to determine the potential influence of bleaching on shear bond strength and enamel colour.

The use of bovine teeth enabled an accordant population substitute for statistical reasons and assured a set of homogenous specimens. Nakamichi et al. [15] reported that the adhesion to human and bovine enamel did not show a significant statistical difference although the mean values were always slightly higher with the latter. This difference, when compared to the human enamel, may be attributed to the larger crystal grain structure and greater lattice defects found in the bovine enamel resulting from rapid growth before and after eruption [16]. The use of bovine material does not provide identical results but does provide a consistent data set for further evaluation and comparative interpretation.

Clinical use must also consider that the human eye does not perceive colour differences of Δ*E* < 1 [13]. Δ*E* > 3 perception is categorized as a measuring error and is unacceptable. Vital teeth, above all, may have different colours, although these differences will be minimal [14].

Hydrogen peroxide in high concentration is recommended for quick results in the shortest time to achieve brightening [14]. Significant results did not occur despite close adherence to the manufacturer's application guidelines. The original bright state of the specimens and insufficient time, 36 hours, to stabilize the new colour between bleaching and colorimetric measurement may offer a possible explanation [13]. The values obtained for L*- and b*- with a small-area spectrophotometer (CM-3500d, Minolta GmbH, Langenhagen, Germany), according to Bosch et al. [14], are significantly lower. The use of a 3 mm fixed-window colour meter (here: 4 mm) and the differences particularly of L*-values, are ascribed to the edge loss of light that diffuses through and emerges at the surface outside of the measurement area.

The analysis of parameter b* indicates a double colour shift for Group II, two-step bleaching and debonding, whereas it was not evident in the single step, Group I. The hydrogen peroxide also removes the organic surface layer in addition to its primary function as a strong oxidizing agent [2].

A sound enamel surface reinforces the opal effect by refracting more of the short wave lengths of light and resulting in a greater perception of the blue colouration [8]. The actual colour shift measured in Group II within this treatment might be due to the timing of the colour measurement following bleaching and assuming that bonding and debonding have no influence on b* values. If indeed this measurement was taken too quickly as previously mentioned, the implication would be that not all the new colour settings were captured at that time. Any remaining changes then would be ascribed to bonding and debonding. A possible extension of the bleaching process in the storage media may also not be excluded.

The slight green shift of debonded and unbleached teeth in Group I evidences primarily a colour shift in a way that colour shifts may also be initiated by the irreversible penetration of prism-like resin tags remaining on debonded sites even after cleanup of the surface area [11].

In this study, bleaching of the enamel prior to bonding seems to provide superior adherence to the treated surface and results in increased bond strength in the bleached group. Bleaching as a preparatory step to resin application is not advised without consideration of the ARI Score results. The predominant score 1 (all of the resin remains on the enamel surface after debonding) may lead to the conclusion that adhesion forces failed, exclusively in the interface located between resin and the bracket base. Cohesive failure can be excluded to a great extent as scores 2 and 3 are not worthy of mention. This was consistent in both groups, proving no relationship to pretreatment, and because of the failed bracket base adherence, no conclusions about absolute bond strength between enamel surface and resin may be made. Another aspect of possible bond strength distortion may be seen in the positioning of specimens in the universal testing machine [17]. The crosshead blade which presses the bonded bracket to failure, in fact, also creates undesirable lever effects.

## 5. Conclusions

Hydrogen peroxide containing bleaching agents is now widely available for private use and creates a probable conflict between dentistry and home treatment. It affects not only the bleaching but also in a greater sense the entire field of aesthetic and regular orthodontic care.

In conclusion, bonding and debonding procedures are not affected by a peroxide pretreatment of enamel. Colour differences are initiated by bleaching as described in this in vitro study. The principal factors that determine bright teeth and an adequate bonding performance have more to do with proper treatment, observing and allowing sufficient time for remineralisation and colour adoption of sound enamel. The debonding of orthodontic devices might lead to colour change as a result of remnant adhesive resin and not appropriate cleaning and polishing procedures.
